# A Move in the Wrong Direction

**Published:** 1898-08

**Authors:** 


### A MOVE IN THE WRONG DIRECTION.

An effort is being made to change the place of meeting of the National Association of Dental Faculties and to meet independently of the National Dental Association. The arguments brought forward to sustain this are that three-fourths of the delegates are from points east of the Mississippi, and that a large number of delegates are not active members of the National and feel very little interest in it. That it would be better to go to Chicago, Indianapolis, Cincinnati, or Louisville.

This effort to separate the place of meeting has been made almost yearly, and has, with one exception, invariably failed. A large proportion of the members of the Faculties are active members of the National Association as at present organized, and feel a deep interest in it. To stop half-way to attend the Association of Faculties and then proceed would be to many an annoyance of such magnitude that it is presumed few would think of attempting the additional journey. To deprive the national organization of the services of these men simply means a reduction of a large portion of its vitality. It must be remembered that Boston, New York, and Baltimore furnish one-fifth of the delegates, who have the longest journeys to make, yet it is assumed that their preference would be to meet where they could enjoy the larger social life which is the result of a combination of associations.

The experiment of separation was tried at Chicago some years ago, and resulted in a very unsatisfactory meeting of the Faculties, owing to the anxiety of the members to hurry the business and start for the meeting of the American Dental Association.

There is no urgent reason for a change from former years, and if put to a vote it should be negatived.

